# Long-Term In Vitro Culture Alters Gene Expression Pattern of Genes Involved in Ontological Groups Representing Cellular Processes

**DOI:** 10.3390/ijms25137109

**Published:** 2024-06-28

**Authors:** Wiktoria Zgórecka, Wiesława Kranc, Małgorzata Blatkiewicz, Kacper Kamiński, Maryam Farzaneh, Artur Bryja, Paul Mozdziak, Paweł Antosik, Maciej Zabel, Marzenna Podhorska-Okołów, Piotr Dzięgiel, Bartosz Kempisty, Dorota Bukowska

**Affiliations:** 1 Department of Anatomy, Poznan University of Medical Sciences, 60-781 Poznan, Polandwkranc@ump.edu.pl (W.K.); 2 Department of Histology and Embryology, Poznan University of Medical Sciences, 60-812 Poznan, Poland; mblatkiewicz@ump.edu.pl (M.B.);; 3 Fertility, Infertility and Perinatology Research Center, Ahvaz Jundishapur University of Medical Sciences, Ahvaz, Iran; maryamfarzaneh1394@gmail.com; 4 Division of Anatomy, Department of Human Morphology and Embryology, Faculty of Medicine, Wroclaw Medical University, 50-368 Wroclaw, Poland; artur.bryja@umw.edu.pl; 5 Physiology Graduate Program, North Carolina State University, Raleigh, NC 27695, USA; pemozdzi@ncsu.edu; 6 Prestage Department of Poultry Science, North Carolina State University, Raleigh, NC 27695, USA; 7 Department of Veterinary Surgery, Nicolaus Copernicus University in Toruń, 87-100 Toruń, Poland; pantosik@umk.pl; 8 Division of Anatomy and Histology, University of Zielona Góra, 65-417 Zielona Góra, Poland; m.zabel@inz.uz.zgora.pl; 9 Division of Histology and Embryology, Department of Human Morphology and Embryology Faculty of Medicine, Wroclaw Medical University, 50-368 Wroclaw, Poland; piotr.dziegiel@umw.edu.pl; 10 Division of Ultrastructural Research, Faculty of Medicine, Wroclaw Medical University, 50-368 Wroclaw, Poland; marzenna.podhorska-okolow@umw.edu.pl; 11 Center of Assisted Reproduction, Department of Obstetrics and Gynecology, University Hospital and Masaryk University, 602 00 Brno, Czech Republic; 12 Department of Diagnostics and Clinical Sciences, Institute of Veterinary Medicine, Nicolaus Copernicus University in Toruń, 87-100 Toruń, Poland; dbukowska@umk.pl

**Keywords:** reproductive biology, cell adhesion, cell migration, intercellular communication, RNA processing

## Abstract

The oviduct provides an optimal environment for the final preparation, transport, and survival of gametes, the fertilization process, and early embryonic development. Most of the studies on reproduction are based on in vitro cell culture models because of the cell’s accessibility. It creates opportunities to explore the complexity of directly linked processes between cells. Previous studies showed a significant expression of genes responsible for cell differentiation, maturation, and development during long-term porcine oviduct epithelial cells (POECs) in vitro culture. This study aimed at establishing the transcriptomic profile and comprehensive characteristics of porcine oviduct epithelial cell in vitro cultures, to compare changes in gene expression over time and deliver information about the expression pattern of genes highlighted in specific GO groups. The oviduct cells were collected after 7, 15, and 30 days of in vitro cultivation. The transcriptomic profile of gene expression was compared to the control group (cells collected after the first day). The expression of *COL1A2* and *LOX* was enhanced, while *FGFBP1*, *SERPINB2*, and *OVGP1* were downregulated at all selected intervals of cell culture in comparison to the 24-h control (*p*-value < 0.05). Adding new detailed information to the reproductive biology field about the diversified transcriptome profile in POECs may create new future possibilities in infertility treatments, including assisted reproductive technique (ART) programmes, and may be a valuable tool to investigate the potential role of oviduct cells in post-ovulation events.

## 1. Introduction

In mammals, the oviduct provides an optimal environment for the final preparation, transport, and survival of gametes, the fertilization process, and early embryonic development [1,2,3,4,5]. Fallopian tubes are narrow passageways which the ova travel from each ovary to the uterus. Oviductal tissue is highly sensitive to the fluctuating levels of sex steroid hormones during different stages of the estrous cycle [6,7]. The processes occurring in the female reproductive system are relatively well described, but the changes on the molecular level are not fully known, because the research is still being conducted and knowledge in this area is expanding. Fertilization usually occurs in the distal third of the fallopian tube which curves over the ovary, and the ampulla contains ciliated epithelial cells with tubal fimbria [5]. The preimplantation phase is filled with crucial events that require effective embryo–maternal interactions involving oviductal secretion and other cellular and molecular interactions [5,8], such as embryonic genome activation during the migration of the embryo through the oviduct [9,10]. The fertilized oocyte undergoes its first cell divisions, and then cells increase their intracellular contacts, leading to compaction (morula stage), blastocoele formation, and cell differentiation [11]. The transcriptomic changes in the oviducts are related to the regulation of embryo implantation and development [12].

Over the past few years, various cell culture models for primary oviductal epithelial cells were established. Monolayer cultures of OECs and 3D culture models [1] are frequently utilized as a model showing interactions of the oviduct with spermatozoa [4] or cumulus–oocyte complexes (COCs) [3,5]. 

Recent studies [13,14,15,16,17] have shown that POECs during in vitro culture may change their morphology and biochemical properties [13,18]. The presented studies used standardized OECs primary in vitro culture methods, which have already been used in the analysis of gene expression related to processes such as “angiogenesis and circulatory system development” [19], “cell cycle”- and “cell death”-related genes [17] and genes linked to cellular proteins defined in Gene Ontology as “maintenance of location”, “maintenance of protein location”, and “maintenance of protein location in cell” [20] and oxygen metabolism [21].

The current results demonstrate more transcripts that may be associated with metabolic regulators, immune modulators, enzymes, and extracellular matrix components, and hence be associated with oviductal early events in maintaining stable oviductal physiology. It is proven that abnormalities in fallopian tubes from infections, surgeries, tumors, or rare congenital malformations may be the cause of ectopic pregnancy [22]. Additionally, ovarian carcinoma is a highly heterogeneous group of diseases because of the different histological subtypes with distinct molecular genetic backgrounds. Nowadays, there is a development in genomic studies focused on the origin of ovarian cancer [23,24,25,26]. Labidi-Galy et al. in 2017 published groundbreaking results which revealed that the development of high-grade serous ovarian carcinoma (HGSOC) is the result of a seeding event from an initial tumor in the fallopian tubes. Serous tubal intraepithelial carcinomas (STICs) might be precursors for most HGSOCs [25]. This discovery has opened a conversation between scientists as to whether ovarian cancer subtypes other than HGSOC, such as low-grade serous cancers and endometrial cancers, may also arise from cells outside of the ovary.

Numerous studies based on in vitro cultures confirmed the important effect of oviductal epithelia and oviductal fluid on early embryonic development in mammals, like sheep, mice, pigs, and cattle [27,28,29,30]. The oviductal microenvironment provides stable conditions, including an optimal temperature, pH, and fluid secretions. It is a place where important embryonic changes occur, like the first mitotic cleavage and embryonic genome activation at the 8-cell blastocyst [31]. Oviductal epithelia are also responsible for the synthesis of embryotropic factors, such as growth factors (EGF, FGF, IGF, TGF) [3,5]. In turn, tubal fluid contains catalase, superoxide dismutase, and glutathione peroxidase to reduce the stress of the embryos from reactive oxygen, and in vivo, it protects the embryo from environmental stress. The oviduct also participates in autoimmunology protection of the embryos by inhibiting the production of antimicrobial peptides and excess protease activity [5].

The continuation of research on primary cultured OECs may be a valuable tool to investigate the original properties of the oviductal epithelium and the potential role of oviduct cells in other biochemical processes and post-ovulation events, including oocyte–oviduct interactions and early embryo development, as well as hormonal actions on reproductive processes occurring within the oviduct [13,32]. The selective transport of embryos through the oviduct indicates a reciprocal interaction of the embryo on gene expression [29].

Therefore, in the present study, the expression profile of genes clustered in GO groups representing cellular processes and interactions, such as “cell adhesion”, “rRNA processing”, “ribosome biogenesis”, “DNA regulation”, “cell migration”, “collagen fibril organization”, “extracellular matrix organization”, “response to viruses”, “positive regulation of transcription from RNA polymerase II promoter”, and others were assessed. The “cell adhesion” Gene Ontology group is associated with the attachment of a cell, either to another cell or to an underlying substrate such as the extracellular matrix, via cell adhesion molecules [33]. Changing the expression of genes related to the adhesion process may be of key importance in the transport of the spermatozoa, oocyte, and zygote [34].

Determining the changes associated with these processes will allow us to understand the mechanism of the fertilization process that takes place in the ampulla of the oviduct. The main objective of this study was to characterize changes in gene expression associated with the regulation of cellular processes such as “cell adhesion”, “cell migration”, “intercellular communication”, “rRNA processing”, “ribosome biogenesis”, and “extracellular matrix organization” occurring in porcine oviductal epithelial cells in primary in vitro culture.

It is assumed that there will be a change in the expression profile of the *COL1A2*, *LOX*, *OVGP1*, *GCNT3*, *RSAD2*, *CHRDL1*, *LUM*, and *EPCAM* genes, which play a key role in processes such as “cell adhesion”, “cell migration”, “intercellular communication”, “rRNA processing”, “ribosome biogenesis”, and “extracellular matrix organization” occurring in the epithelial cells of porcine oviductal cells.

## 2. Results

Oviduct cells were collected after 7, 15, and 30 days of cultivation to assess changes in the direction of gene expression profiles. The transcriptomic profile of gene expression was compared to the control group (24-h).

**Figure 1:**
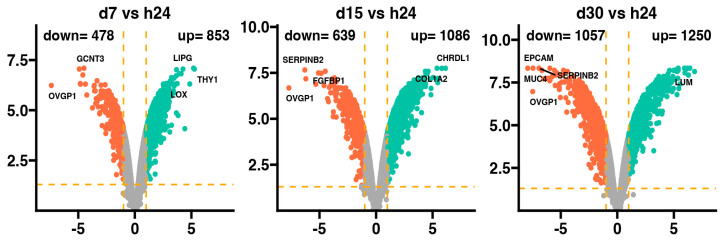
Volcano plots of differentially expressed genes in the d7, d15, and d30 experimental groups compared to the h24 control group. Cut-off values shown on the graph as orange dashed lines were obtained based on parameters |fold change| = 2; *p*-value = 0.05. Red dots represent genes with downregulated expression, while green dots—upregulated expression. The exact number of differentially expressed genes in each comparison is shown in the upper part of the graph. The five most overexpressed genes are labeled with their names. Two biological replicates were performed for each experiment.

The general profile of the transcriptome changes is shown in the Figure 1, where dots represent the mean gene expression. With respect to the assumed cut-off criteria for differentially expressed genes (|fold change| = 2, and *p*-value = 0.05), we demonstrated 478 genes with downregulated expression and 853 with upregulated expression in 7-day vs. 24-h, 639 genes with downregulated expression and 1086 with upregulated expression in 15-day vs. 24-h, and 1057 genes with downregulated expression and 1250 with upregulated expression in 30-day vs. 24-h comparisons.

In the 7-day and 24-h comparisons of the experiment, the genes with downregulated expression profile include *GCNT3* and *OVGP1*, with overexpression of the *LIPG*, *THY1*, and *LOX* genes. In the 15-day vs. 24-h comparisons, the most downregulated genes were *SERPINB2*, *FGFBP1*, and *OVGP1*, while the most overexpressed were the *CHRDL1* and *COL1A2* genes. Meanwhile, at 30 days vs. 24 h, it has been indicated that *EPCAM*, *SERPINB2*, *MUC4*, and *OVGP1* were downregulated, while only *LUM* was upregulated. Interestingly, it has been observed that only the expression of the *OVGP1* gene was reduced in all analyzed groups compared to the 24-h group.

**Figure 2:**
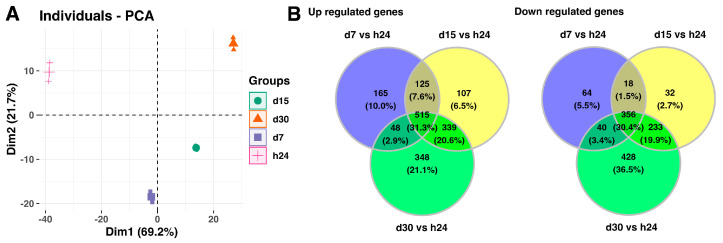
(**A**) Principal component analysis (PCA) plot of the first two components of the filtered microarray data set. (**B**) Venn diagrams show genes with upregulated (left panel) and downregulated (right panel) expression shared among different analyzed groups.

Next, the analysis includes full data sets comprising the analyzed groups, and it is presented in Figure 2. The principal component analysis (PCA) indicates the variation between the different biological groups. The first principal component (Dim1) explains 69.2% of variations between analyzed groups, while the second principal component accounts for 21.7% of the variance. The PCA plot shows that the groups of the control and day 7 of the experiment, scattered in the left region of the plot, were separated from the groups of day 15 and day 20 of the experiment, which were scattered in the right region of the plot (Figure 2A). Furthermore, the Venn diagrams indicates that a high number of the analyzed genes are commonly expressed in the experimental conditions, 515 genes with upregulated expression (31.3%) and 356 genes with downregulated expression (30.4%), compared to the 24-h point of the experiment (Figure 2B).

A list of the top 20 genes with the highest (10 genes) and lowest (10 genes) expression fold change at 7, 15, and 30 days of the experiment compared to the 24-h point of the experiment is presented in Table 1, Table 2, and Table 3, respectively.

The fold change values of the top ten overexpressed genes at the 7-day vs. 24-h point (Table 1) ranged from 38.75 (*THY1*) to 12.88 (*FMOD*), while the downregulated expression of the top ten genes ranged from −12.17 (*TXNIP*) to −166.19 (*OVGP1*).

The compassion of gene expression between the 15-day and 24-h points of the experiment (Table 2) indicates that the ten genes with the most enhanced expression ranged from 70.42 (*CHRDL1*) to 24.48 (*CDH11*), meanwhile the fold change of the ten top genes with downregulated expression ranged from −18.51 (*LOC100524999*) to −209.47 (*OVGP1*).

The fold change values for the top ten overexpressed genes at the 30-day vs. 24-h point of the experiment (Table 3) ranged from 114.07 (*LUM*) to 41.92 (*LOC106508700*), whereas for the genes with downregulated expression, the fold changed ranged from −61.38 (*CLDN7*) to −243.04 (*EPCAM*).

In summary, it appears that the expression of *COL1A2* and *LOX* was enhanced while *FGFBP1*, *SERPINB2*, and *OVGP1* were downregulated at the 7-, 15-, and 30-day points when compared to the 24-h control.

Furthermore, the analysis also includes a functional annotation of differentially expressed genes evaluated using the Database for Annotation, Visualization, and Integrated Discovery (DAVID) bioinformatics tool with the GO BP Direct database.

**Figure 3:**
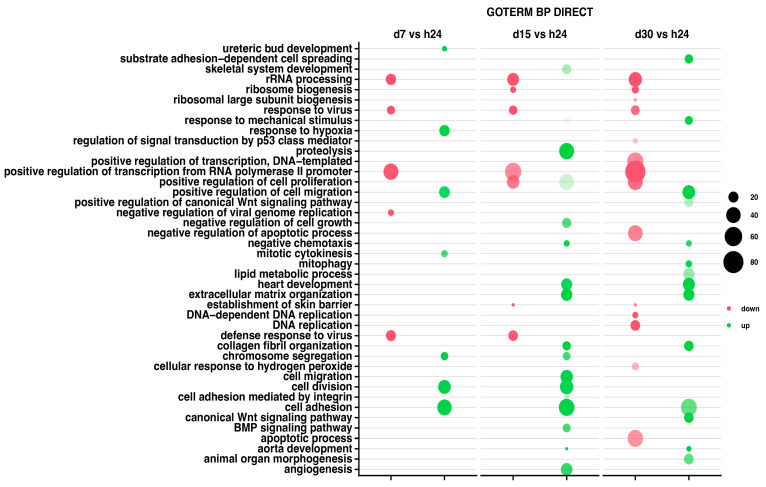
Bubble plot showing differentially expressed gene sets in DAVID GO BP DIRECT database. Each column represents corresponding gene sets in comparison groups d7 vs. h24; d15 vs. h24; d30 vs. h24, respectively. Red bubbles represent downregulated expression and green bubbles represent upregulated expression gene sets. The size of the bubble reflects the number of different genes in a particular GO term. Higher bubble transparency means closer proximity of *p*-value to 0.05. The parameters used for the cut-off criteria were *p*-value with correction < 0.05, number of genes in set >2.

The relevant GO ontological groups with adjusted *p*-values below 0.05 and N per group > 2 are presented in Figure 3, where comparison of the 7-day vs. 24-h point of experiment shows seven activated and five inhibited GO BP terms. In other experimental groups, it has been shown that a 15-day vs. 24-h comparison showed 17 activated and 7 inhibited GO BP terms, while a 30-day and 24-h of experiment comparison indicates 14 activated and 14 inhibited GO BP terms.

For day 7 of the experiment, the most inhibited GO term was “rRNA processing” (n = 21, *p* = 1.005 × 10^−13^). Aside from this process, the GO terms related to the biological response to viruses were also inhibited (mostly, response to viruses n = 14, *p* = 5.98 × 10^−8^), being similar at day 15 of the experiment (n = 15, *p* = 3.09 × 10^−7^). At the day 30 of the experiment, aside from rRNA processing (n = 34, *p* = 6.86 × 10^−18^), there has been also indicated an inhibition of the apoptotic (n = 45, *p* = 9.52 × 10^−5^) and cell development processes (ribosome biogenesis n = 12, *p* = 2.91 × 10^−7^).

The 7-day to 24-h of the experiment comparison indicates mostly the GO term “response to hypoxia” (n = 21, *p* = 2.52 × 10^−6^), as well as chromosome segregations (n = 13, *p* = 6.6 × 10^−6^) and cell adhesion (n = 40, *p* = 8.39 × 10^−6^). In the second group, at day 15 of the experiment, the most activated GO terms were proteolysis (n = 43, *p* = 6.11 × 10^−8^) and extracellular matrix organization (n = 25, *p* = 8.27 × 10^−8^). Also, the analysis of GO terms at day 30 confirmed the activation of cellular organization processes (collagen fibril organization, n = 18, *p* = 1.36 × 10^−9^).

For all analyzed groups, we observed some similarities in GO terms expression; “rRNA processing” was mostly inhibited across all analyzed groups. Moreover, “response to viruses” and “positive regulation of transcription from RNA polymerase II promoter” were also inhibited in all analyzed groups. Nevertheless, only “cell adhesion” has been activated across all groups.

**Figure 4:**
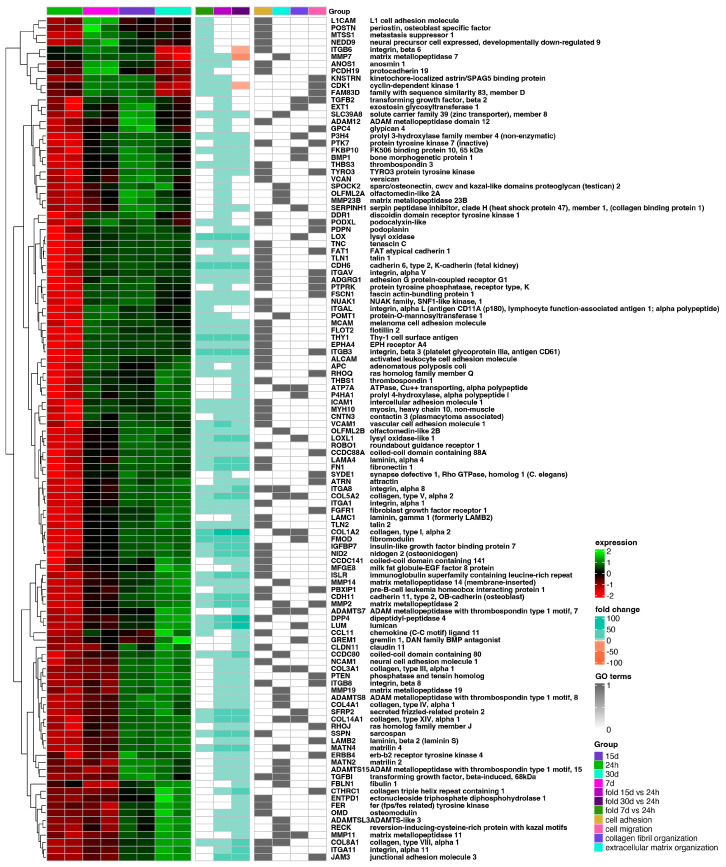
Heatmap of expressed gene clusters in 24 h control group and d7, d15, and d30 experimental groups. Leftmost heat map shows scaled levels of gene expression in each study group (green = 2, black = 0, red = −2). The middle heatmap shows the fold change of the gene expression in comparison to the 24 h control group. The right heatmap highlights genes belonging to the four most significantly enriched ontological groups (black = present, white = absent).

The hierarchic clustering of differentially expressed genes in all analyzed groups has been shown as a heatmap and presented in Figure 4. The figure shows mean expression values, normalized expression values, and fold changes between compared groups. Genes belonging to the most significantly enriched ontological groups (lowest adjusted *p*-value) are shown as dark squares. Expression values are scaled by rows and presented as colors and ranges.

**Figure 5:**
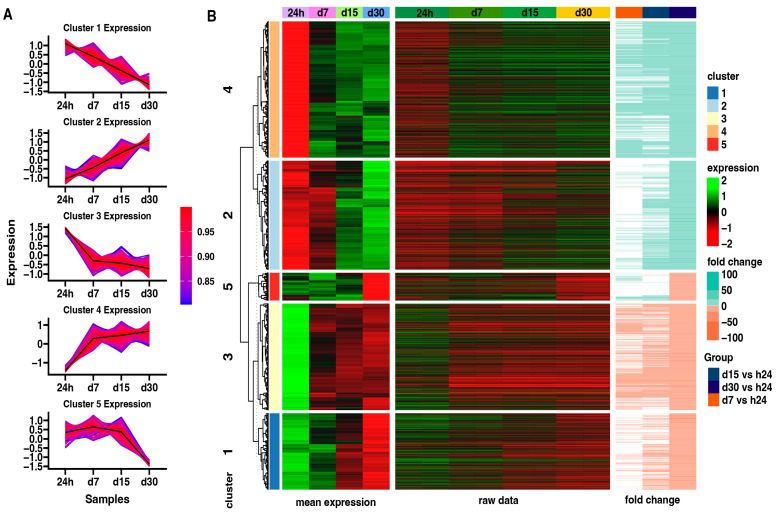
(**A**) Clustering of differentially expressed genes. Centroid values are shown as black lines. Each line represents an individual gene, where the color corresponds to the level of correlation to the centroid values according to the color scale. (**B**) Heatmap of differentially expressed genes in all analyzed groups divided into five gene clusters. Leftmost heat map illustrates mean expression of genes grouped in five clusters in 24 h control group and in each study group: d7, d15, d30 (green = 2, black = 0, red = −2). The rightmost heatmap shows the fold change of the gene expression compared to the 24 h control group.

Subsequently, an analysis of the expression patterns in each group using the K-means clusterization algorithm has been made (Figure 5). First, the optimal number of clusters has been determined using the sum of squared error (SSE) approach, with an increasing number of clusters. By using this algorithm, five clusters was determined as the optimal number (Figure 5A). The centroid values and core gene expression set have been determined for each cluster. Cluster 1 contains the genes whose expression is highest at the beginning of the experiment and decreases with time. Cluster 2 shows the opposite expression profile to Cluster 1. Next, Cluster 3 presents the genes expressed mostly at the beginning of the experiment, with a similar reduction in expression from 7 to 30 days of the experiment. Cluster 4 contains the genes inhibited at the beginning of the experiment, with high enhancement since day 7 till the end of the experiment. Cluster 5 presents genes expressed highly from the 24-h to 15-day points of the experiment, with a significant decrease at the 30-day point.

**Figure 6:**
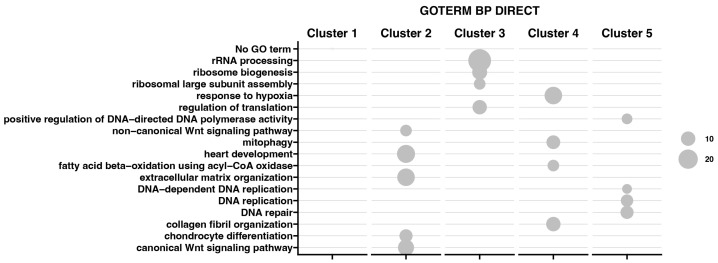
Bubble plot representing enrichment of genes in GO groups divided among five gene clusters. The size of the bubble corresponds to the number of genes assigned to the GO BP terms.

Moreover, the results obtained by K-means clusterization have been transcribed into GO BP terms by DAVIVD and presented in Figure 6 as a bubble plot for all clusters. It has been indicated that for Cluster 1, none of the GO processes has been assigned. Cluster 2 is mainly associated with ECM organization and the canonical and non-canonical Wnt signaling pathway. Cluster 3 contains genes responsible for rRNA processing, ribosome biogenesis, and regulation of translation. Custer 4 represents genes in response to hypoxia, mitophagy, and collagen fibril organization, while Cluster 5 is responsible for DNA replication, repair, and the positive regulation of DNA-directed DNA polymerase activity.

## 3. Discussion

Unlike the ovary and uterus, fallopian tube physiology is less understood for its contribution to reproduction. The challenge for scientists is to identify appropriate in vitro models to facilitate the study of early embryo–maternal communication. In this study, only genes associated with cell molecular pathways such as “rRNA processing”, “ribosome biogenesis”, “DNA regulation”, “cell adhesion”, “cell migration”, “collagen fibril organization”, “response to viruses”, “extracellular matrix organization”, “positive regulation of transcription from RNA polymerase II promoter”, “canonical Wnt signalling pathway”, and “proteolysis” were examined in the analysis, which may play role in gametes and fertilized egg transport. The mentioned processes may be regulated in the fallopian tube during fertilization and the passage of the embryo from oviduct to uterus [34]. For instance, the cell–cell adhesion molecules in gamete transport facilitate the attachment of the sperm to the oviductal epithelium. It is also known that cumulus–oocyte complexes (COCs) express adhesion molecules, such as E-cadherin and N-cadherin. The adhesion between the extracellular matrix of the cumulus cells (CCs) and oviductal cells correlates with successful oocyte pick-up and subsequently the transfer of COCs to the infundibulum and oviduct [35,36]. Additionally, adhesion molecules have a role in initiating the compaction state through implantation [36].

Microarray assay analysis allows the identification of different groups of genes and their interrelationships. It has expanded the field of research of the valid transcriptomic factors in the porcine oviductal epithelium. During the in vitro culture of POECs, changes in gene expression were analyzed at different time intervals: 1 (24 h), 7, 15, and 30 days of cultivation.

Standardization of the cell culture is crucial for stable in vitro conditions of cell culture for further study. The characterization of the passage method, stable temperature conditions, and the well-defined composition of the culture medium provide high-quality experimental reproducibility in mammalian cell culture. This approach makes it possible to evaluate the expression profile of individual genes in porcine oviductal epithelial cells.

The results indicate changes in the gene expression of defined gene clusters during the in vitro cell culture. Cluster 2 includes genes related to extracellular matrix organization and the non-canonical and canonical Wnt signaling pathway, and it shows the lowest expression at the beginning and an increase with time. Cluster 3 consists of genes with a similar reduction in expression from 7 to 30 days of the experiment—genes linked to “rRNA processing”, “ribosome biogenesis”, “ribosomal large subunit assembly”, and “regulation of translation”. Cluster 4 contains genes belonging to the groups “response to hypoxia”, “mitophagy”, “fatty acid beta-oxidation using acyl-CoA oxidase”, and “collagen fibril organization”, and they were inhibited at the beginning of the experiment, with high upregulation at day 7 till the end of the experiment. Cluster 5 presents genes expressed highly from the 24-h to 15-day interval of the experiment, with significant limitation at the 30-day interval. Genes in this group related to the apoptotic process, DNA replication, and repair can be included, such as *CDK1* (Figure 4). It may suggest the limited functions of repair mechanisms due to aging and the accumulation of damage in cells over time after crossing the 30th day of long-term in vitro primary cultivation [37,38,39,40].

Most of the studies focus on oviductal fluid and proteins secreted into it [41,42,43]. Banliat et al. identified 56 proteins involved in embryo–maternal interactions in the bovine oviduct by mass spectrometry, such as annexins (ANXA1, ANXA2, ANXA4), OVGP1, and PYGL [41]. A reduced expression of the oviduct-specific glycoprotein gene (*OVGP1*) was revealed in all analyzed groups compared to the 24-h control. It is well described in the literature that *OVGP1* belongs to the group of MUC proteins, and it is a main constituent of oviductal fluid in early embryonic enhancement [44,45]. The roles of oviductal glycoprotein 1 relate to the maintenance of sperm viability and motility and sperm capacitation in the oviduct [46] and oocyte zona pellucida stability, including the process of acquiring resistance to proteolytic digestion during oviductal transit [47]. Recently, Nelson et al. [48] conducted an RNAseq analysis of granulosa cells (GCs) collected from mice and suggested that a reduced expression of the OVGP1 gene in GCs could have an impact on pre-ovulation processes such as cumulus expansion and oocyte maturation.

Another mucin-type encoding gene was downregulated at the 30th day—MUC4, a membrane-bound mucin, a family member of highly glycosylated proteins, which play an important role in the protection of epithelial cells and have been implied in epithelial renewal and differentiation [49].

In the group of genes studied at day 7 of culture, one was also significantly downregulated. *GCNT3* is a gene directly linked with mucin-type biosynthesis, because it codes a glycosyltransferase that can synthesize all known mucin beta 6 N-acetylglucosaminides [50].The reduced expression of all mucin-type or mucin-related protein-encoding genes through the in vitro culture may be connected with the absence of an embryo in the in vitro long-term culture conditions of the cells, because it has been suggested that mucins are expressed during the peri-implantation period in the uterus in vivo [51].

*RSAD2* is a member of the S-adenosyl-L-methionine (SAM) super family of enzymes and a crucial factor in cellular antiviral response and innate immune signaling, and it was significantly downregulated on day 7 [52]. A transcriptional profiling of the ovine urine endometrium conducted by Song et al. [53] showed an increased transcript of *RSAD2* with other related genes between days 12 and 16 of pregnancy, but not of the estrous cycle. Schmaltz-Panneau’s experimental data [54] of the in vitro co-culture of early bovine embryos with bovine oviduct epithelial cells (BOECs) experiment show an increased expression of *RSAD2*, as opposed to our results relating to the single culture of porcine OECs.

As we indicated earlier, *FGFBP1*, *SERPINB2*, and *SERPINB5* expression was also downregulated. *FGFBP1* encodes a fibroblast growth factor binding protein 1, and it plays a critical role in various cell mechanisms and cell–cell signaling, such as proliferation, differentiation, and migration, by binding to fibroblast growth factors [55,56,57]. Lee et al. suggested that the primary function of *FGFBP1* may be connected to sustaining cellular survival throughout embryogenesis [58]. *SERPINB2*, known also as plasminogen activator inhibitor type 2 (PAI)-2, is predicted to be involved in the negative regulation of endopeptidase activity and is one of the most upregulated proteins after cellular stress [59]. Its expression is acutely upregulated in pregnancy and inflammation an infection state [60], and was also demonstrated during the in vitro culture of porcine granulosa cells (pGCs) [39], while a decreased expression *SERPINB2* was noted during the culture of porcine OECs [17]. Serpin 5, also known as maspin (encoded by *SERPINB5*), is commonly described as a tumor and metastasis suppressor [61,62]. Its expression, present in human mammary epithelial cells and downregulated during cancer progression [63], has been described. The main biological roles of maspin are cell adhesion, migration, control of gene expression and oxidative stress response [64]; these functions are therefore due to the expression of *SERPINB5*.

From the genes analyzed in day 15 of cultivation, two genes were significantly upregulated—*CHRDL1* and *COL1A2*. *COL1A2* (collagen type I alpha 2 chain) belongs to the “extracellular matrix organization”. *CHRDL1* is another gene contributing to the neuronal differentiation of neural stem cells in the brain and may play a role in embryonic bone formation. This gene, belonging to the “BMP signalling pathway” GO group, antagonizes the function of bone morphogenetic protein 4 (BMP4) by binding to it and preventing its interaction with receptors. Due to this role, CHRDL1 has an important role in regulating retinal angiogenesis through the modulation of BMP4 actions in endothelial cells [65]. Wang et al. [66] suggested that *CHRDL1*, with the interaction of other genes (TWSG1 and CHRD) in ovarian granulosa cells, may modulate the intra-ovarian functions of the TGF-β superfamily members, such as the control of progesterone production. A similar conclusion was indicated in earlier research conducted on porcine oocytes [67]. The upregulation of *CHRDL1* may be connected to its function in early stages of folliculogenesis and oogenesis regulation in pigs. The observed overexpression of *CHRDL1* in this study may be connected with the preparation of oviductal cells for maternal–oocyte interactions. The expression of gene-encoding lysyl oxidase was upregulated in all the analyzed intervals in the presented study. *LOX* is related to the GO group “collagen fibril organization”, which links to extracellular matrix formation in pOECs.

Interestingly, up to day 30 of in vitro cell culture, the most overexpressed gene was *LUM* (lumican), which is similar to the results of Kedem et al. [68] with mRNA samples from mural (MGCs) and cumulus granulosa cells. *LUM* expression was induced in high-density cell cultures in a confluence-dependent manner. Its role might be described as a new potential ovulatory marker during the preovulatory period up until ovulation, as well as in endometriosial infertility [68,69].

*EpCAM* was the gene that was the most downregulated up to the 30-day interval. EpCAM is an antigen expressed in most normal epithelial cells, numerous stem and progenitor-type cells, and most carcinomas, and is highly overexpressed in cancer-initiating cell types [70]. In carcinoma cells, EpCAM takes part in cell adhesion, proliferation, migration, stemness, and epithelial-to-mesenchymal transition (EMT) [71,72], showing its multifunctional transmembrane protein role. Additionally, it plays an important role in embryonic stem cells proliferation and differentiation, and with other molecules is responsible for maintaining the murine embryonic stem cell’s phenotype [73]. The decreased expression of *EpCAM* at day 30 of in vitro culture in our study might be in response to EMT induction, because it seems that the changes in cell morphology require the downregulation of EpCAM and E-cadherin [74,75].

This work was performed on the basis of standard methods used in molecular biology. The results presented are a contribution to an even better understanding of the processes occurring in the fallopian tube. Those may in the future contribute to a better understanding of pathological processes occurring within the female reproductive system.

## 4. Materials and Methods

### 4.1. Tissue Collection

Porcine oviducts were collected at the abattoir from local crossbred landrace gilts (n = 20) at an age of approximately nine months and weight of 98 kg. Pigs were kept in the same conditions, in accordance with the standards of breeding and feeding of pigs. The animals were kept in accordance with the commonly acknowledged technology for this particular group, with attention paid to their well-being. Animals were checked daily for estrus behavior and were slaughtered after reaching the anestrus phase of the estrus cycle. The oviducts were excised within 30 min of slaughter. The collected tissues were immediately transported to the laboratory and kept in an isolated container. The use of animals for the purpose of scientific research complied with all the relevant Polish national and European Union regulations and institutional policies for the care and use of animals.

### 4.2. Primary Long-Term Cell Culture of Porcine Oviductal Epithelial Cells (POECs)

In this study, the establishing of the long-term POECs culture in vitro was based on the earlier described protocol by Kulus et al. [13]. Oviducts were washed twice in Dulbecco’s phosphate buffered saline (DPBS; 137 mM NaCl; 27 mM KCl; 10 mM Na_2_HPO_4_; 2 mM KH_2_PO_4_; pH 7.4). Epithelial cells were surgically removed using sterile blades and then enzymatically digested for 1 h at 37 °C with 1mg/mL collagenase I (Sigma Aldrich, St. Louis, MO, USA) in Dulbecco’s modified Eagle’s medium (DMEM; Sigma Aldrich, St. Louis, MO, USA, Merck KGaA, Darmstadt, Germany). The cell suspension obtained from this digestion was filtered through a 40 µm strainer to remove blood and aggregated cells and centrifuged for 10 min at 200× *g* at room temperature (RT). Next, the resulting pellet was washed in PBS and centrifuged again at 200× *g* for 10 min at RT. After rinsing with PBS, the POECs were incubated for another 10 min at 37 °C with 0.5% Trypsin/EDTA (Sigma Aldrich, St. Louis, MO, USA, Merck KGaA, Darmstadt, Germany). After a time, the reaction was stopped with fetal calf serum (FCS; Sigma-Aldrich, St. Louis, MO, USA), and cells were filtered and centrifuged again at 200× *g* for 10 min at RT. The final cell pellet was resuspended in DMEM supplemented with 10% FCS, 100 U/mL penicillin, 100 µg/mL streptomycin, and 1 µg/mL amphotericin B, and the cells were cultured up to 30 days at 37 °C in a humidified atmosphere of 5% CO_2_. The cells’ vitality used for primary culture was rated 90%. Initially, the cells proliferated very slowly, but after 7 days of culture, they reached similar parameters of growth and density. The culture medium was changed every three days. Once the OECs reached a confluency of around 70–80%, they were passaged to another culture dish at a seeding density of 2 × 10^4^ cells/cm^2^. Cells in the culture were detached from the bottom of the culture bottle by 1–2 min incubation with 0.5% Trypsin/EDTA (Sigma Aldrich, St. Louis, MO, USA, Merck KGaA, Darmstadt, Germany). The proliferation rate of the cells corresponded to the days of cell collection. The cultures were passaged three times. Samples of cells from the 7th day of culture were collected as a culture sample defined as a short-term culture; the cells on 15th day of culture showed the effects of the first passage, and the cells on the 30th day of culture were comparable after three passages. The dynamic changes in cell morphology were monitored under an inverted microscope (relief contrast) throughout the in vitro primary cultivation of the cells.

### 4.3. RNA Isolation from Porcine Oviductal Epithelial Cells (POECs)

The total RNA from the POECs were isolated using TRI Reagent (Sigma Aldrich, St. Louis, MO, USA) and an RNeasy MinElute Cleanup Kit (Qiagen, Hilden, Germany). The RNA for transcriptome study was collected from two independent replicates for each experimental variant: (1) control—24 h, (2) 7-day, (3) 15-day, and (4) 30-day interval of experiments. Each replicate contained pooled RNA from three independent experiments. The amount of total mRNA was determined from measuring the optical density (OD) at 260 nm, and the RNA purity was estimated using the 260/280 nm absorption ratio on a NanoDrop™ spectrophotometer (Thermo Fisher Scientific, Inc., Waltham, MA, USA). Samples purity was obtained if the ratio was >1.8. RNA integrity and quality were checked on a Bioanalyzer 2100 (Agilent Technologies, Inc., Santa Clara, CA, USA). The resulting RNA integrity numbers were between 8.5–10. The RNA in each sample was diluted to a concentration of 100 ng/μL with an OD260/OD280ratio of 1.8–2.0. For each sample, 100 ng RNA was taken for the microarray analysis.

### 4.4. Microarray Expression Study

A cDNA synthesis was made from total RNA (100 ng) during a two-step reaction, biotin labeling and fragmentation according to the manufacturer’s protocol (GeneChip^®^ WT Plus Reagent Kit, Affymetrix, Santa Clara, CA, USA). Next, the biotin-labeled cDNA was hybridized to the Affymetrix^®^ PorGene 1.1 ST Array Strip (45 C/20 h) and stained by the Affymetrix GeneAtlas Fluidcs Station of GeneAtlas System. The Imaging Station of the GeneAtlas System (Affymetrix, Santa Clara, CA, USA) has been used for microarrays scanning. Moreover, the Affymetrix GeneAtlas Operating System was used for analyzing the performed results and evaluation of the quality of gene expression data in accordance with the software’s quality control criteria.

### 4.5. Microarray Data Analysis

The microarray study was carried out according to the previously described procedure [76,77]. All bioinformatics analyses have been performed by using BioConductor software (release 3.15, https://bioconductor.org, accessed on 1 April 2024) with relevant BioConductor packages, functioning as an extension of the R programming language (v4.2.1; R Core Team 2022).

For the normalization, background correction, and calculation of the expression values of the analyzed genes, the Robust Multiarray Average (RMA) normalization algorithm implement in the “Affy” library was applied [78]. To show the total number of up- and downregulated genes, a principal component analysis (PCA) of the filtered data set was performed and visualized using the “factoextra” library [79].

The DAVID (Database for Annotation, Visualization, and Integrated Discovery) bioinformatics tool has been used for the functional annotation and clusterization of differentially expressed genes (DEGs) [80]. The established cut-off criteria for DEGs were based on the differences in the absolute value from an expression fold change greater than 2. Further, the expressed genes were assigned to relevant GO terms, with the subsequent selection of significantly enriched GO terms using the GO BP DIRECT database. Gene symbols of differentially expressed genes were uploaded to DAVID by the “RDAVIDWebService” BioConductor package [81], where DEGs were assigned to relevant Gene Ontology (GO) terms, with the subsequent selection of significantly enriched GO terms from the GO BP FAT database. The *p*-values of selected GO terms were corrected using the Benjamini–Hochberg correction; DEGs from each comparison were visualized by the hierarchic clustering of differentially expressed genes as a heatmap using the “ComplexHeatmap” library [82,83].

### 4.6. mRNA Co-Expression Analysis—Clustering of mRNA Data

A set of mRNAs expression data whose expression was significantly regulated in at least one of the compared pairs were selected for analysis. To determine the optimal number of clusters, a repeatedly calculated sum of squared error (SSE) measurement with an increasing number of clusters was applied. The K-means algorithm was used for the clustering of mRNA expression profiles (according to the manual from: https://2-bitbio.com/2017/10/clustering-rnaseq-data-using-k-means.html (accessed on 1 April 2024). Clustering was performed on the average expressions from each experimental group using the “kmeans” core R function.

For each cluster, the centroid values and core mRNA sets were determined. Core mRNAs were generated by filtering the fitting level of mRNAs expression to centroid values, where the mRNA expression profile displayed a high correlation to centroids for a given cluster (correlation > 0.8). The mean expression, normalized expression values, and fold changes for the core mRNA of each cluster were visualized as a heatmap using the “Complexheatmap” package [83]. Next, the DAVID bioinformatic tool was used to identify the functional annotation and clusterization of the target genes [84,85].

## 5. Conclusions

Each molecular factor identified during in vitro studies, and which could be potentially significant and used as a supplement in assisted reproductive techniques, must be evaluated by the analysis of signaling pathways. Currently, the domestic pig (*Sus scrofa*) is one of the best models used by researchers because of its close association with human physiology.

The general changes in transcriptomic profiles in POECs genes belong to ontological groups related to different processes occurring within epithelial oviductal cells. Some of the genes are specific to the reproductive system and may have a potential role as novel biomarkers of porcine oviductal epithelial cells, but most corresponded to cellular processes in all type of cells. The current results represent an essential next step in understanding the regulatory processes occurring in the mammalian oviduct.

## Figures and Tables

**Table 1:** A list of the top 20 most regulated genes from the comparison group d7 vs. h24, including 10 genes with the highest and 10 genes with the lowest fold change. Values were obtained using following parameters: |fold change| > 2, *p*-value < 0.05.

**Gene Symbol**	**Gene Name**	**Fold Change**
*THY1*	Thy−1 cell surface antigen	38.75
*LIPG*	lipase, endothelial	36.55
*LOX*	lysyl oxidase	29.05
*ANKRD1*	ankyrin repeat domain 1 (cardiac muscle)	21.40
*UNC45B*	unc−45 homolog B (C. elegans)	19.12
*LOC106508700*	protein prune homolog 2−like	17.16
*COL1A2*	collagen, type I, alpha 2	15.57
*TMSB15A*	thymosin beta 15a	14.21
*UBE2QL1*	ubiquitin−conjugating enzyme E2Q family−like 1	13.45
*FMOD*	Fibromodulin	12.88
*TXNIP*	thioredoxin interacting protein	−12.17
*SLC28A3*	solute carrier family 28 (concentrative nucleoside transporter), member 3	−13.42
*OASL*	2−5−oligoadenylate synthetase−like	−19.01
*GPX2*	glutathione peroxidase 2	−20.67
*CD274*	CD274 molecule	−22.60
*SERPINB2*	serpin peptidase inhibitor, clade B (ovalbumin), member 2	−24.27
*FGFBP1*	fibroblast growth factor binding protein 1	−27.95
*RSAD2*	radical S−adenosyl methionine domain containing 2	−28.39
*GCNT3*	glucosaminyl (N−acetyl) transferase 3, mucin type	−30.10
*OVGP1*	oviductal glycoprotein 1, 120 kDa	−166.91

**Table 2:** A list of the top 20 most regulated genes from the comparison group d15 vs. h24, including 10 genes with the highest and 10 genes with the lowest fold change. Values were obtained using following parameters: |fold change| > 2, *p*-value < 0.05.

**Gene Symbol**	**Gene Name**	**Fold Change**
*CHRDL1*	chordin−like 1	70.42
*COL1A2*	collagen, type I, alpha 2	56.38
*AGTR1*	angiotensin II receptor, type 1	44.11
*LOX*	lysyl oxidase	43.85
*FMOD*	Fibromodulin	41.89
*THY1*	Thy−1 cell surface antigen	32.74
*SULF1*	sulfatase 1	29.09
*COL14A1*	collagen, type XIV, alpha 1	28.88
*FBN1*	fibrillin 1	27.55
*CDH11*	cadherin 11, type 2, OB−cadherin (osteoblast)	24.48
*LOC100524999*	placenta−specific gene 8 protein	−18.51
*OASL*	2−5−oligoadenylate synthetase−like	−19.59
*CD274*	CD274 molecule	−22.51
*GPX2*	glutathione peroxidase 2	−24.24
*MUC1*	mucin 1, cell surface associated	−28.79
*GCNT3*	glucosaminyl (N−acetyl) transferase 3, mucin type	−34.27
*ANKRD22*	ankyrin repeat domain 22	−40.07
*FGFBP1*	fibroblast growth factor binding protein 1	−74.13
*SERPINB2*	serpin peptidase inhibitor, clade B (ovalbumin), member 2	−78.88
*OVGP1*	oviductal glycoprotein 1, 120 kDa	−209.47

**Table 3:** A list of the top 20 most regulated genes from the comparison group d30 vs. h24, including 10 genes with the highest and 10 genes with the lowest fold change. Values were obtained using following parameters: |fold change| > 2, *p*-value < 0.05.

**Gene Symbol**	**Gene Name**	**Fold Change**
*LUM*	Lumican	114.07
*CHRDL1*	chordin−like 1	85.62
*AGTR1*	angiotensin II receptor, type 1	78.21
*PRLR*	prolactin receptor	73.13
*DPP4*	dipeptidyl−peptidase 4	71.86
*FBN1*	fibrillin 1	62.85
*COL1A2*	collagen, type I, alpha 2	61.33
*LOX*	lysyl oxidase	46.65
*LOC102164159*	myozenin−2−like	43.49
*LOC106508700*	protein prune homolog 2−like	41.92
*CLDN7*	claudin 7	−61.38
*SPINT1*	serine peptidase inhibitor, Kunitz type 1	−62.17
*APOBEC1*	apolipoprotein B mRNA editing enzyme, catalytic polypeptide 1	−63.28
*AQP9*	aquaporin 9	−69.25
*FGFBP1*	fibroblast growth factor binding protein 1	−90.37
*SERPINB5*	serpin peptidase inhibitor, clade B (ovalbumin), member 5	−101.70
*SERPINB2*	serpin peptidase inhibitor, clade B (ovalbumin), member 2	−121.38
*MUC4*	mucin 4, cell surface associated	−175.03
*OVGP1*	oviductal glycoprotein 1, 120 kDa	−179.30
*EPCAM*	epithelial cell adhesion molecule	−243.04

## Data Availability

The data presented in this study are available on request from the corresponding author.
